# Some Remarks on the Nature and Treatment of Cancers

**Published:** 1784

**Authors:** 


II. *Some Remarks on the nature and treatment of Cancers. Communicated in a letter to Dr.* Simmons, *by* Robert White, *M. D. Phyſician at Eye in Suffolk.*

NO diſeaſe to which the human ſpecies is ſubject carries with it ſo formidable an appearance, or is productive of ſuch dreadful conſequences, as that which is called a Cancer. It has ever been the reproach of the medical art, and the moſt learned and experienced of the profeſſion feffion have employed their time and attention to but little purpofe towards perfecting its cure. Various remedies for this difeafe have indeed at different times been publifhed, but it is a well-known and melancholy truth, that not one of them has yet been able to ftand the teft of experience. Of late years our attention has been much excited by the means of cure propofed by Baron Storck.——Mercury, bark, and cicuta have been often tried in this country, according to the rules prefcribed by that learned phyfician, but have not produced the effect which the perufal of his ingenious publication on the fubject gave us reafon to expect. I have had an opportunity, however, of feeing many flattering inftances of relief in the ulcerated ftate of cancer, from a continued ufe of the abovementioned medicines; and am firmly perfuaded, that his method of treating the difeafe would often be productive of the defired effect, provided the ichorous, corrofive quality of the matter, which lodges from time to time upon the furface of the fore, could be corrected, and the abforption of that matter be prevented, in a certain degree. This additional plan I have endeavoured to put in practice, and have had the happinefs to find it fucceed in two defperate cafes. In a third it has failed; failed; but from the nature of the parts affected, which were the cheek, tongue, and fauces, I had very little reafon to expect even the temporary relief which vifibly followed its ufe.

The method I purfue to prevent abforption, and correct the acrimony of the difcharge, is by fumigation and poultice; the latter of thefe alone, has been attended with all the fuccefs that could be defired, in a cafe to which I was called fome time fince, of an aged perfon, whofe breaft had been long difeafed, but for a year or two before had difcharged at times an ichorous matter, and healed again with little trouble: at length it inflamed, gangrened, and floughed intirely off. The fore healed in about ten weeks, and remained fo not long fince. A common poultice, with dry lint, and lint lightly fpread with cerate, were the only applications made ufe of during the whole procefs of cure. No medicine was taken internally, except a gentle laxative.

As foon as I have had an opportunity of making another trial or two of this method, upon the ulcerated cancer, in which ftate it is particularly to be applied, I fhall beg leave to lay before the public, through the medium of that ufeful work the Medical Journal, the whole of the procefs, and the event; although it may not be attended with with that positive success which I am so sanguine as to hope for, still I flatter myself that it will serve as a hint to those of superior skill and understanding, to turn their thoughts more readily to a subject of so great importance.

It is the doctrine of the day that this disease is originally local. From the favourable change which has immediately followed the application of the above-mentioned remedies, I am induced to think that it is so even in the ulcerated state; and that when the habit is tainted in a high degree, it becomes so from the fœtid, sanious discharge being absorbed, and contaminated with a pre-existent acrid state of the juices. This may be stiled matter of opinion only. Still, however, I am not as yet so confident in that opinion, as to reject the use of bark, mercury, and opium.

Pity it is that in this disease, the opportunity of procuring relief is so little minded, and so often lost. In the simple, detached, indurated state of cancer, excision is attended with little pain, no danger, and perfect success. Terror and false hope are in cases of this sort too often suffered to get the better of reason and resolution. Even men of great judgment in the profession have sometimes flattered themselves and

 their their patients with the hopes of avoiding an operation, which, in the early ſtage of the diſeaſe, produces ſo much comfort and ſecurity. To obviate ſuch miſchief, I beg leave to preſent the following practical hints, which, if duly attended to, may tend to ſhorten the progreſs of an evil, which, if neglected, muſt be productive of the worſt conſequences:

1. In its infant ſtate, when the tumor is round, ſmooth, and not hard to the touch, the diſeaſe *often* yields to an alterative courſe.

2. When the tumor is become large, round, ſmooth, and in ſome degree indurated, it *ſeldom* gives way to ſuch a mode of treatment.

3. When the tumor is hard and unequal, and attended with pricking pain, it *ſcarcely ever* admits of relief from ſuch means; and I believe *never* when it has attained what may be conſidered as a fourth ſtage, that is, when the tumor is of a ſtony hardneſs and very unequal, attended with acute ſhooting pains. In this latter ſtage of the diſorder, when the breaſt begins to loſe its natural colour, and the nipple is drawn in, the knife ſhould be ſubmitted to without heſitation; and indeed from duly conſidering the progreſs of the diſeaſe as ſpecified in the above hints, I am convinced that the eaſieſt, easiest, safest, and most proper periods for extirpation are in the second and third stages.

Eye, Suffolk, Dec. 8, 1783.

